# Interrelationship Between Contractility, Protein Synthesis and Metabolism in Mantle of Juvenile Cuttlefish (*Sepia officinalis*)

**DOI:** 10.3389/fphys.2019.01051

**Published:** 2019-08-23

**Authors:** Simon G. Lamarre, Tyson J. MacCormack, Émilie Bourloutski, Neal I. Callaghan, Vanessa D. Pinto, José P. Andrade, Antonio V. Sykes, William R. Driedzic

**Affiliations:** ^1^Département de Biologie, Université de Moncton, Moncton, NB, Canada; ^2^Department of Chemistry and Biochemistry, Mount Allison University, Sackville, NB, Canada; ^3^Faculty of Applied Science and Engineering, Institute of Biomaterials and Biomedical Engineering, University of Toronto, Toronto, ON, Canada; ^4^Translational Biology and Engineering Program, Ted Rogers Centre for Heart Research, Toronto, ON, Canada; ^5^Centro de Ciências do Mar do Algarve, Campus de Gambelas, Universidade do Algarve, Faro, Portugal; ^6^Department of Ocean Sciences, Memorial University, St. John’s, NL, Canada

**Keywords:** anaerobic metabolism, cycloheximide, glucose, iodoacetic acid, jetting, octopine

## Abstract

Young juvenile cuttlefish (*Sepia officinalis*) can grow at rates as high as 12% body weight per day. How the metabolic demands of such a massive growth rate impacts muscle performance that competes for ATP is unknown. Here, we integrate aspects of contractility, protein synthesis, and energy metabolism in mantle of specimens weighing 1.1 g to lend insight into the processes. Isolated mantle muscle preparations were electrically stimulated and isometric force development monitored. Preparations were forced to contract at 3 Hz for 30 s to simulate a jetting event. We then measured oxygen consumption, glucose uptake and protein synthesis in the hour following the stimulation. Protein synthesis was inhibited with cycloheximide and glycolysis was inhibited with iodoacetic acid in a subset of samples. Inhibition of protein synthesis impaired contractility and decreased oxygen consumption. An intact protein synthesis is required to maintain contractility possibly due to rapidly turning over proteins. At least, 41% of whole animal *Ṁ*O_2_ is used to support protein synthesis in mantle, while the cost of protein synthesis (50 μmol O_2_ mg protein^–1^) in mantle was in the range reported for other aquatic ectotherms. A single jetting challenge stimulated protein synthesis by approximately 25% (2.51–3.12% day^–1^) over a 1 h post contractile period, a similar response to that which occurs in mammalian skeletal muscle. Aerobic metabolism was not supported by extracellular glucose leading to the contention that at this life stage either glycogen or amino acids are catabolized. Regardless, an intact glycolysis is required to support contractile performance and protein synthesis in resting muscle. It is proposed that glycolysis is needed to maintain intracellular ionic gradients. Intracellular glucose at approximately 3 mmol L^–1^ was higher than the 1 mmol L^–1^ glucose in the bathing medium suggesting an active glucose transport mechanism. Octopine did not accumulate during a single physiologically relevant jetting challenge; however, octopine accumulation increased following a stress that is sufficient to lower Arg-P and increase free arginine.

## Introduction

Cephalopods have a short life span and a semelparous reproductive pattern which gives them the reputation of living in the “fast lane.” Early juvenile cuttlefish (*Sepia officinalis*) grow at a rate of up to 12% body mass per day (Sykes et al., 2006, 2014). To support this rapid growth rate, cuttlefish mantle muscle has an unusually high fractional rate of protein synthesis compared to most fish and other invertebrate species (Lamarre et al., 2016). The cost of protein synthesis accounts for a major proportion of overall metabolic rate in marine organisms and is typically estimated between 11 and 42% of total oxygen consumption (Fraser and Rogers, 2007). To our knowledge, the cost of protein synthesis has never been measured in cephalopods but both protein synthesis and metabolic rates are highly sensitive to fasting in cuttlefish (Lamarre et al., 2016); this observation suggests that protein synthesis accounts for a substantial proportion of basal oxygen consumption in cephalopods as well.

Cuttlefish and other cephalopods utilize jet propulsion as a means of locomotion to avoid predators and other disturbances. Jet propulsion is energetically inefficient relative to the undulatory swimming used by fish and perhaps the best demonstration of this is provided by O’Dor and Webber (1986), who determined that the net cost of transport (J Kg^–1^ m^–1^) is 3–4.5 times higher in squids compared to trout. This means that in order to travel the same distance, cephalopod mantle muscles have to work much harder than fish swimming muscles. For squids, the magnitude of this differential is not equal for all body sizes as jetting efficiency scales allometrically, with juveniles being more efficient than adults (O’Dor and Hoar, 2000; Bartol et al., 2008). In mammalian skeletal muscles, secondary metabolic demands are also imposed on the tissue during recovery from intense exercise in the form of an increase in rates of protein synthesis (Wong and Booth, 1990a, b; Biolo et al., 1995; Phillips et al., 1997). To our knowledge, the occurrence of this phenomenon has never been examined in cephalopods. If rates of protein synthesis are already maximized to support growth in juvenile cuttlefish, frequent jetting events may require a redistribution of resources away from growth and toward recovery, prolonging the time to maturity. If they do maintain scope to further increase rates of protein synthesis in the mantle muscle following a jetting event, it may impose yet another energetic demand on their already highly solicited aerobic metabolism.

Jetting events result from powerful contractions of the mantle muscle. In squid and cuttlefish forced to exercise to exhaustion, activity is supported by anaerobic means involving the transphosphorylation of arginine-phosphate and the breakdown of glycogen to generate ATP, with octopine generated as an end-product (Storey and Storey, 1979; Pörtner et al., 1993). In contrast, in scallops forced to swim to exhaustion or exposed to acute hypoxia, little octopine accumulates during the challenge; the main increase in octopine concentration is instead observed during the subsequent recovery period (Gäde et al., 1978; Grieshaber, 1978). This delayed increase in octopine production may be caused by the low affinity of octopine dehydrogenase (ODH) for pyruvate, which does not reach a sufficient concentration to activate the enzyme until anaerobic glycolysis has been upregulated (van Os et al., 2012). If protein synthesis is activated to facilitate recovery from exercise, the additional energetic demands it imposes must be supported by either aerobic or anaerobic metabolism. Characterizing which specific means of generating ATP are utilized will allow a better understanding of growth and development of cuttlefish and inform aquaculture practice with respect to dietary requirements.

Here, we tested the hypothesis that the protein synthesis is stimulated following muscle contractions simulating a jetting event in the mantle of early juvenile cuttlefish. We examined electrically stimulated mantle muscle strip preparations and integrated information from different levels of organization to develop a better understanding of the energetics in mantle of young animals. Preparations were forced to contract at a frequency observed under normal jetting activity (Trueman, 1980) and were treated with cycloheximide (CHX), which interferes with the translocation step in protein synthesis, and iodoacetic acid (IAA), that inhibits glycolysis at the level of glyceraldehyde-3-phosphate dehydrogenase. The contractile protocol was subsequently applied to determine rates of tissue oxygen consumption (*Ṁ*O_2_), rates of protein synthesis, glucose utilization, and octopine production. The data set allowed a calculation of a protein energy budget and provides new insights into the subtle importance of glucose trafficking in mantle muscle.

## Materials and Methods

### Ethics Statement

All the procedures were approved by the CCMAR Animal Welfare Committee (ORBEA CCMAR-CBMR) and the Direcção-Geral de Alimentação e Veterinária (DGAV) of the Portuguese Government, according to National (Decreto-Lei 113/2013) and the EU legislation (Directive 2010/63/EU) on the protection of animals used for scientific purposes. In addition, protocols were approved by institutional Animal Care Committees at Université de Moncton (UdeM-18-02) and the Memorial University of Newfoundland. Procedures were only applied to live animals by authorized users.

### Animal Husbandry and Euthanasia

Experiments were done during May 2018, at CCMAR’s Ramalhete Aquaculture Station (Ria Formosa, Portugal – 37°00′22.39″N; 7°58′02.69″W). Cuttlefish (*S. officinalis*) were reared from hatching to the juvenile stage, in a 1500 L round fiberglass black tank in an open seawater system, according to the latest culture technology described in Sykes et al. (2014). All procedures were applied to juveniles of a F2 captive stock. Animals were 3–8 weeks post hatch (final part of the hatchling stage and entering the juvenile stage) with a mass of 1.1 ± 0.04 (SEM) g (*N* = 41). Temperature, salinity, and dissolved oxygen saturation (DO_2_) were measured daily, at 9 h 30 min, in the stock tank. Both temperature and DO_2_ were measured with a VWR DO220 probe, while salinity was measured with a VWR EC300 salinity meter. Water temperature was 20.8 ± 1.14 (SD) °C, salinity was 34.6 ± 0.71 (SD) g L^–1^ and DO_2_ was 101.0 ± 1.60 (SD)%. Cuttlefish were fed thawed grass shrimp (*Palaemonetes varians*) *ad libitum* on a daily basis during the experimental period.

In terminal experiments, animals were euthanized in seawater containing 10% ethanol (Sykes et al., 2012). Time to cessation of ventilation was ≈2 min and, afterward, assurance of death was achieved by ventral bisection of the brain and severing of the ventral nerve and optical lobes (Lewbart and Mosley, 2012). Specimens were splayed open to expose two wings of mantle tissue which was subsequently sampled and carefully skinned.

### Whole Animal Oxygen Consumption

Resting *Ṁ*O_2_ was assessed using an automated intermittent flow respirometry system (Q-Box AQUA, Qubit Systems, Kingston, ON, Canada). Animals were removed from their holding tank, weighed, and quickly transferred to a 215 mL cylindrical respirometry chamber (4 cm diameter). The chamber was housed in a darkened 10 L reservoir of continuously aerated seawater at 20°C. Animals were transferred into the respirometry chamber at ≈16:00 h, and *Ṁ*O_2_ was monitored overnight. Oxygen levels were recorded at 15 min intervals throughout the experiment: 5 min with the system in closed loop followed by a 10 min flush cycle between each reading. Animals were housed in the respirometer for 8–16 h, and *Ṁ*O_2_ returned to baseline levels within the first 2–3 h after transfer into the system. The resting *Ṁ*O_2_ values reported here represent the mean of at least measurements taken in the final 2 h of the experiment. *Ṁ*O_2_ was corrected for any background level.

### Experimental Design and Incubation Media

**FIGURE 1:**
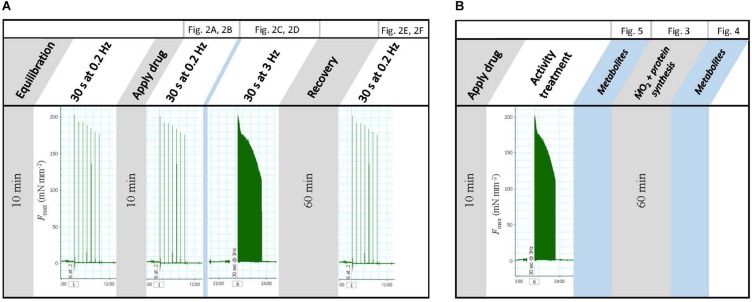
Schematic of experimental design. **(A)** Contractility study. **(B)** *Ṁ*O_2_, protein synthesis, and metabolite levels.

All experiments, other than whole animal *Ṁ*O_2_ measurements, involved isolated preparations of either mantle strips (oxygen consumption and contractile studies) or mantle sheets (protein synthesis and metabolite levels), which were prepared as described below. The overall experimental design is shown in Figure 1 and discussed in detail in the appropriate sections that follow. Basic incubation medium consisted of 0.22 μm filtered sea water supplemented with 1 mmol L^–1^ glucose. The one exception to this was an experiment to test the immediate impact of high frequency stimulation on metabolite levels in which the bathing medium did not contain glucose. Medium was further supplemented with either 25 μmol L^–1^ CHX to impair protein synthesis, with the other preparation receiving an equivalent volume of DMSO as a vehicle control, or 1 mmol L^–1^ IAA to impair glycolysis.

### Contractile Performance of Mantle Strips

Contractile performance of mantle preparations was measured to assess the impact of inhibition of protein synthesis and glycolysis and to set the experimental parameters for metabolic studies. Animals were euthanized as described above and paired circumferential sections (i.e., perpendicular to the long axis of the animal) of ≈8 mm long by 1 mm wide were cut from the widest (funnel-side) side of the ventral mantle. The skin was gently removed by blunt dissection and a 6-0 silk suture was tied around one end of each muscle strip. The opposite end of the muscle strip was then clamped between two platinum electrodes in a double-walled 20 mL Plexiglas chamber and the suture thread fastened to a calibrated isometric force transducer (Harvard Apparatus, South Natick, MA, United States). The chambers contained basic incubation medium. The medium was continuously aerated and maintained at 20.0 ± 0.01°C using a recirculating water bath. Strips were stimulated to contract via field stimulation across the platinum electrodes using a Grass SD9 stimulator (Grass Technologies Inc., Warwick, RI, United States) and were gently stretched to optimal peak tension development prior to commencing each experiment, as in previous studies (Milligan et al., 1997). Force transducers were interfaced to a PowerLab 4/26 (ADInstruments, Colorado Springs, CO, United States) data acquisition system and data were recorded and analyzed using associated LabChart 8 software.

After mounting, preparations were allowed to equilibrate without stimulation for 10 min before being triggered to contract six times, as a measure of basal performance, at a frequency of 0.2 Hz using 100 V (nominal), 5 ms duration square wave pulses. One of the paired preparations was then treated with either CHX or IAA. Preparations were incubated for an additional 10 min, stimulated six times at 0.2 Hz to assess treatment effects on basal contractility, and then stimulated at 3 Hz for 30 s for a total of 90 contractions to simulate a maximum jetting event. The latter stimulation frequency corresponds to the reported maximum jetting frequency of *S. officinalis* (Trueman, 1980) and the 30 s duration of the challenge reflects the approximate time to 50% loss of maximum force production assessed in preliminary preparations. Following the maximum jetting protocol, muscles were left unstimulated for 1 h to recover before being forced again to contract six times at 0.2 Hz.

Tension measurements were normalized to absolute stress using the nominal cross-sectional area of the preparation and an assumed muscle density of 1.06 g cm^–3^ (Layland and Altringham, 1995), as previously described (Shiels et al., 2010). Each data point represents the mean of at least three representative contractions. Muscle performance was calculated as the change in peak tension (*F*_*max*_) compared to tension developed under the initial six contraction response that served as the hallmark. *F*_*max*_ following the simulated jetting protocol was quantified from the final three contractions in the 30 s stimulation train.

### Oxygen Consumption, Protein Synthesis, and Metabolite Levels During Recovery From Jetting

Rates of oxygen consumption and protein synthesis were determined during the recovery period and metabolite levels after the recovery period following the simulated maximum jetting challenge. The same mantle sheet preparations provided enough tissue mass for measurements of protein synthesis and metabolites; whereas, *Ṁ*O_2_ studies were from mantle strips prepared as above.

#### Oxygen Consumption

The rate of oxygen consumption was measured in paired preparations of mantle strips with a mass of 14.96 ± 3.43 mg. Following isolation, strips were maintained for 10 min in either basic medium (*N* = 5) or medium containing CHX (*N* = 4) or IAA (*N* = 4). Thereafter, one strip was immediately placed in the respiration chamber. The second strip was induced to contract at 3 Hz for 30 s (90 contractions); field stimulation was again applied with 100 V square wave pulses of 20 ms duration with a switch in polarity at 15 s. Following contraction, the strip was immediately placed in the respirometry chamber equilibrated with the same incubation medium. The system consisted of a pair of 1 mL respiration chambers fitted with freshly calibrated Clark type polarographic electrodes and magnetic stirrers (OX1LP, Qubit Systems Inc., Kingston, ON, Canada). The temperature was maintained at 20.0 ± 0.01°C using a recirculating water bath. Oxygen concentration was recorded at a rate of 1 Hz using Logger Pro 3.12 (Vernier Inc., Beaverton, OR, United States). Once tissue O_2_ consumption decreased the O_2_ concentration below 6.8 mg L^–1^, the medium was aerated for approximately 20 s, until it reached saturation (oxygen saturation was considered to equal 7.2 mg L^–1^ in 35 g L^–1^ seawater at 20.0°C). O_2_ concentration was recorded this way for up to 60 min. Between 6 and 12 *Ṁ*O_2_ measurements per mantle strip were obtained. After correcting for the background O_2_ consumption (average of 35.9 ± 15.1% of total O_2_ consumption) *Ṁ*O_2_ is reported in nmol O_2_ g^–1^ min^–1^. The relationship between *Ṁ*O_2_ and time was then fitted using an exponential decay function. This function was then used to integrate the total O_2_ consumption for a period of 60 min which we report as μmoles O_2_ g^–1^ of mantle.

#### Protein Synthesis

For measurements of protein synthesis and metabolite levels, mantle sheets were prepared by isolating mantle as described above, and dividing it into two pieces with a mass of 112.00 ± 3.00 mg. Each sheet preparation was then immediately placed into basic incubation medium. Following a 10 min incubation in media with glucose alone or supplemented with CHX or IAA, one of the paired mantle preparations was induced to contract as described above (*N* = 6 for all conditions). The two mantle sheets were then immediately transferred into two 15 mL centrifuge tubes each containing 2 mL of fresh incubation medium for 1 h. The incubation medium was supplemented with 0.75 mmol L^–1^ phenylalanine and 0.75 mmol L^–1^ deuterated phenylalanine (ring-D5-phenylalanine, Cambridge Isotope Laboratories Inc., Tewksbury, MA, United States). Oxygen concentration was maintained constant by gently bubbling the medium with air. Following the incubation period, the mantle sheets were delicately blotted dry to remove excess incubation medium and flash frozen in liquid nitrogen. An aliquot of the incubation medium was frozen for later glucose and octopine analysis.

The rate of protein synthesis (*ks*) was determined by measuring the incorporation of deuterated phenylalanine into protein using gas chromatography and mass spectrometry (GC-MS) as previously described (Lamarre et al., 2015, 2016). Briefly, ≈10 mg of mantle sheet was homogenized in 0.2 mol L^–1^ perchloric acid (PCA) using a sonicating homogenizer (Q55 Sonicator, Qsonica Inc., Newtown, CT, United States). The homogenate was centrifuged at 10,000 × *g* for 5 min at 4°C. The supernatant was then transferred into a clean tube and frozen as it contained the free phenylalanine pool. The protein pellet was washed three times by resuspending it in 0.2 mol L^–1^ PCA and centrifuging as above. The protein pellet was then hydrolyzed in 6 mol L^–1^ HCl at 110°C for 18 h. Phenylalanine was extracted from the free amino acid pool and the protein-bound pool using solid phase extraction (Bond Elut C18, Agilent Inc., Santa Clara, CA, United States). The final eluate was evaporated to dryness and stored at 4°C until GC-MS analysis. The extracted amino acids were derivatized using pentafluorobenzyl bromide as an alkylating agent and analyzed in the GC-MS as described in Lamarre et al. (2015). The system was composed of an Agilent gas chromatograph (model 7890B) interfaced with a single quadrupole mass selective detector (MSD 5977B). Peak detection and integration were performed using MassHunter (Version B07.01 SP2, Agilent). *ks* (% day^–1^) was calculated using $k_{s}=100\times \frac{S_{b}}{S_{a}}\times \frac{1440}{t}$, where *S*_*b*_ and *S*_*a*_ are the deuterated phenylalanine enrichment of the protein-bound and free pools, respectively; *t* is the incorporation time in min and 1440 is the conversion from min to day.

#### Metabolite Analysis

Tissue preparation for analysis of octopine, arginine, and glucose followed standard protocols previously used with *S. officinalis* mantle (Storey and Storey, 1979; Capaz et al., 2017). Mantle was homogenized in 6% cold PCA. Following centrifugation at 10,000 × *g* for 10 min, the supernatant was neutralized with 2 mol L^–1^ KHCO_3_ and recentrifuged prior to analysis. Octopine and glucose were also determined in incubation media of isolated mantle preparations. All assays were conducted using a microplate reader. Octopine and arginine concentration were determined based on the mmol L^–1^ extinction coefficient of 6.22 for NADH at 340 nm and the calculated pathlength (Capaz et al., 2017). For assay purposes, ODH, that catalyzes the conversion of pyruvate + L-arginine + NADH + H^+^ to octopine + NAD^+^ + H_2_O was purified from scallop muscle as described in Capaz et al. (2017). Octopine concentration was assessed in 100 mmol L^–1^ TRIS, 10 mmol L^–1^ NAD+, pH 9.3. Following addition of excess ODH the reaction was monitored for 2 h to ensure it ran to completion. Mantle extracts from a large *S. officinalis* (≈500 g) were stimulated to tetanus to accumulate a high amount of octopine; serving in this way as a positive control and ensuring that the assay conditions were not limiting. Arginine was measured in media containing 50 mmol L^–1^ imidazole, 4 mmol L^–1^ pyruvate, 0.4 mmol L^–1^ NADH, and excess ODH, pH 7. Tissue arginine content was calculated from standard curves. An attempt was made to determine the level of arginine phosphate following acid hydrolysis (Langer et al., 1976) as successfully applied to jumbo squid (Seibel et al., 2014). In our hands the procedure yielded only qualitative information. Glucose was determined with an assay kit: D-Glucose GOD-POD (NZYTech, Lisbon, Portugal).

#### Impact of Jetting on Metabolite Levels

A limited experiment was also conducted to assess the immediate effect of high frequency electrical stimulation on mantle muscle metabolite levels. Paired mantle sheets were prepared as above. In this instance, the mantle sheets were incubated in 0.22 μm filtered seawater without any further additions or in media containing glucose and IAA. The experimental design was based on noted high tissue levels of glucose in the major study and was intended to assess if glucose was called upon during the fast contraction challenge. A second pair of preparations was treated with IAA to inhibit glycolysis and to potentially gain further insight into the role of intracellular glucose during contraction.

### Data Analysis and Statistics

Data presented in text and figures are expressed as mean ± standard error. Difference in contractile performance between control and either CHX or IAA treated preparations was assessed with an unpaired *t*-test. For mantle O_2_ consumption, rate of protein synthesis, and metabolite levels, two-way ANOVAs with repeated measures were used to assess differences between treatments. Rate of glucose and octopine uptake or release into the incubation media was assessed with a one sample *t*-test versus a theoretical value of zero. GraphPad Prism was used for statistical analysis and statistical significance was set at *p* < 0.05 in all cases.

## Results

Mantle muscle accounted for 28.2 ± 1.16% and head 33.6 ± 1.44% of total body mass (*N* = 8). Whole animal *Ṁ*O_2_ was 5.47 ± 0.46 μmol g^–1^ h^–1^ (*N* = 6).

### Contractility of Mantle Muscle

Isometrically contracting mantle muscle strips remained functionally viable beyond the duration of the experimental challenges reported here. Average peak tension (*F*_*max*_) development following the initial 10 min equilibration period was 104 ± 18 mN mm^–2^ (*N* = 22) and in concurrent studies, a number of preparations exhibited *F*_*max*_ values in excess of 300 mN mm^–2^ (data not shown). As such, the preparation is considered to be robust and a suitable model for both contractile and metabolic studies.

**FIGURE 2:**
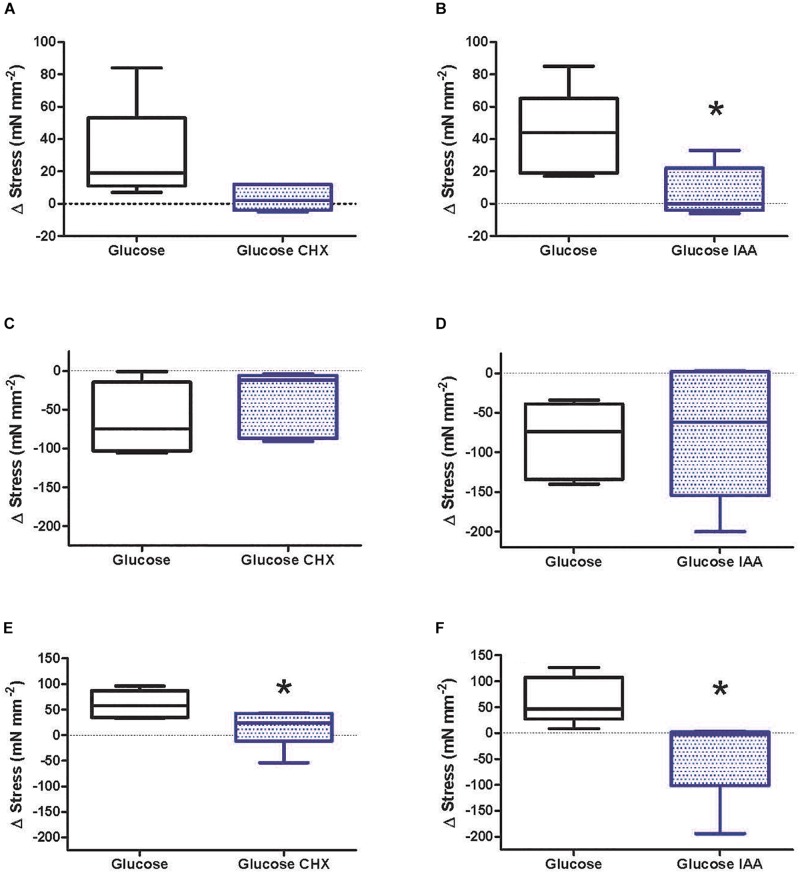
Relative peak stress development by isometrically contracting mantle muscle strips from cuttlefish (*Sepia officinalis*). Preparations were initially stimulated to contract six times at 0.2 Hz. Stress developed during this period served as the level at which all further challenges were related. All experiments involved paired preparations. Bathing media contained glucose (open bars) plus additional 25 μM CHX (stippled bars; left panels) or additional 1 mM IAA (stippled bars; right panels). Panels **(A,B)** – Δ stress development following a 10 min rest followed by a second wave of six contractions at 0.2 Hz. Panels **(C,D)** – Δ stress development following contractions for 30 s at 30 Hz [immediately following **(A,B)**]. Panels **(E,F)** – Δ stress development following 1 h rest [following **(C,D)**]. ^∗^Significant difference in response of matched preparation in media containing glucose alone relative to preparations in media with either additional CHX or IAA. *N* = 6 for CHX experiments and *N* = 5 for IAA experiments. Box and whisker plots show the range and median value.

Tension development in the second wave of six contractions increased following the 10 min unstimulated incubation period in control preparations by approximately 35 mN mm^–2^ (Figures 2A,B; open bars), suggesting the initial 10 min acclimation may not have been sufficient for full recovery. The post rest potentiation was significantly reduced in strips exposed to IAA relative to controls (*p* = 0.036; Figure 2B). A similar pattern of *F*_*max*_ inhibition was noted in muscles treated with CHX, but the effect was not statistically significant (*p* = 0.113) (Figure 2A).

All muscles were able to pace at 3 Hz with no increase in resting tension or evidence of tetanus. Most preparations were able to sustain full *F*_*max*_ for the first ≈45 contractions in the simulated maximum jetting protocol (3 Hz for 30 s) before steadily decaying to values 10–75% of initial after 90 contractions. No significant effects of CHX or IAA treatment relative to controls were noted during this challenge (Figures 2C,D).

Control preparations more than fully recovered from the jetting protocol after a 1 h rest period, exhibiting *F*_*max*_ ≈ 50% higher than hallmark initial levels. Preparations treated with CHX to block protein synthesis failed to recover to the same extent as paired control muscles (*p* = 0.027), although *F*_*max*_ levels did return to pre-exercise levels (Figure 2E). Inhibiting glycolysis with IAA abolished this potentiation of *F*_*max*_ (*p* = 0.044) and preparations failed to fully recover from the challenge (Figure 2F).

### Oxygen Consumption During Recovery From Simulated Jetting

**FIGURE 3:**
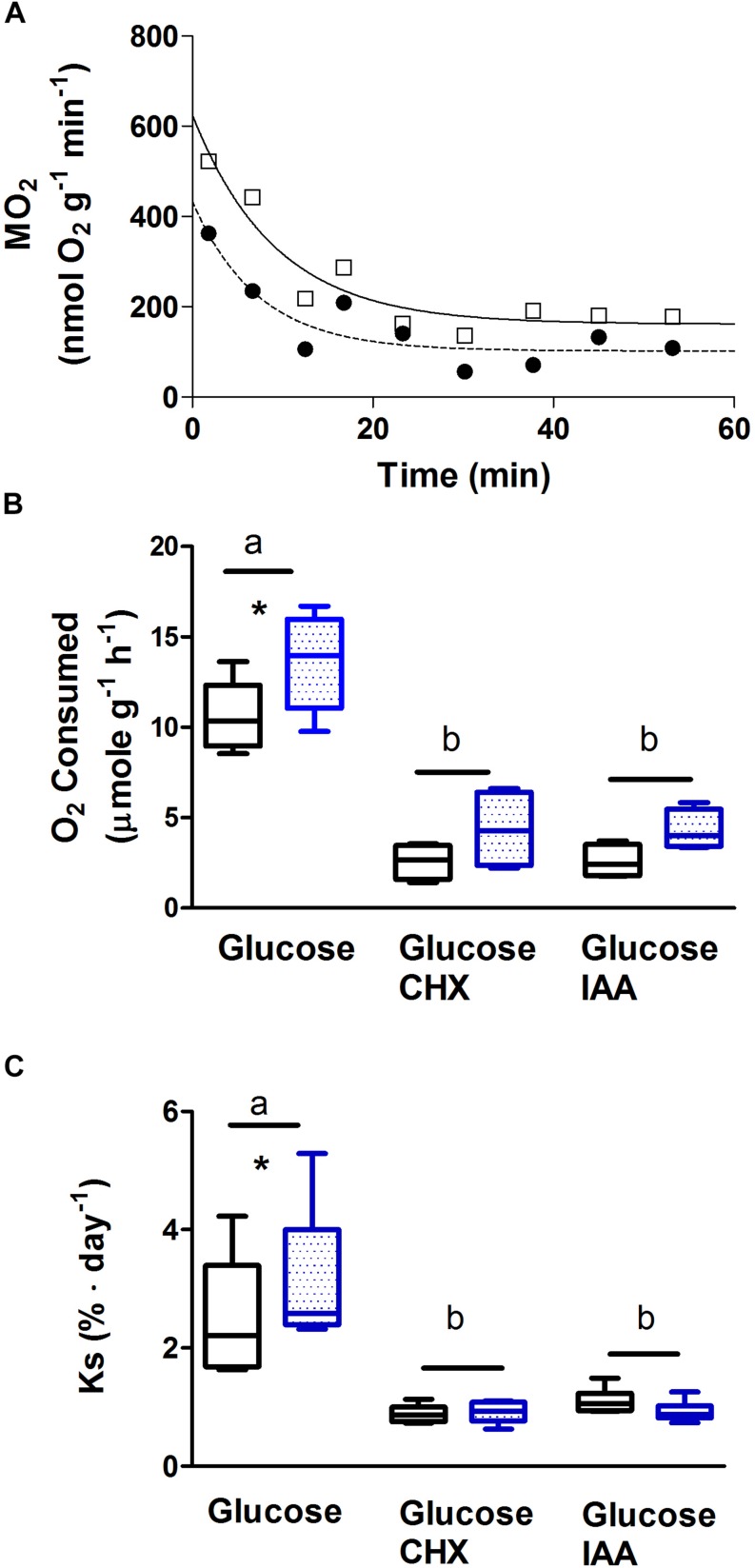
Rate of oxygen consumption and protein synthesis by isolated mantle muscle strips from cuttlefish (*Sepia officinalis*). One of two paired mantle strips was stimulated to contract for 30 s at 3 Hz simulating jetting behavior. Oxygen consumption and protein synthesis was then measured during the recovery period. **(A)** Representative trace of rate of oxygen consumption. The curve with squares and solid line represents oxygen consumption by a mantle preparation that had been forced to contract; the dashed line with circles is the matched unstimulated paired preparation. **(B)** Average rate of oxygen consumed over a 40–60 min period following contraction. **(C)** Rate of protein synthesis measured over a 1 h period following contraction. Open bars represent unstimulated preparations; stippled bars indicate preparations that were stimulated. Text below *x*-axis shows additions to media. For CHX and IAA additions, preparations were incubated for 10 min prior to stimulation, during stimulation, and throughout the post contraction incubation period. For *Ṁ*O_2_ measurements *N* = 7 for glucose; *N* = 4 for CHX and IAA. For protein synthesis *N* = 6 in all cases. Values with different letters are significantly different. ^∗^Significant difference between unstimulated and stimulated preparations. Box and whisker plots show the range and median value.

*Ṁ*O_2_ was determined for isolated mantle strips. One of two paired mantle preparations was subjected to stimulation and thereafter incubated for 1 h in various media. This challenge was similar to that reported in Figures 2E,F in the contractile study. Figure 3A shows a representative response for an individual preparation incubated in basic medium. All preparations showed initial elevations in *Ṁ*O_2_ presumably in part due to oxygen limiting conditions and/or elevated energy demand at some point in setting up the tissue from dissection through the pre-incubation and stimulation periods to measurements of *Ṁ*O_2_ of the incubates. The total oxygen consumed during the incubation period, normalized to 1 h, is presented in Figure 3B. The total oxygen consumed was significantly higher in preparations incubated with glucose alone than with either CHX or IAA included in the media. This was the case for analysis within unstimulated and stimulated preparations. Total oxygen consumed was significantly (30%) higher in preparations that had been stimulated (13.6 ± 1.2 μmol g^–1^ h^–1^) to contract prior to the incubation period than unstimulated (10.5 ± 0.9 μmol g^–1^ h^–1^) preparations when incubated in medium containing glucose alone. This trend was also observed when CHX or IAA was included in the media.

### Protein Synthesis During Recovery From Simulated Jetting

One of two paired mantle sheets was subjected to stimulation as above and thereafter incubated for 1 h in various media. Figure 3C shows the mean rate of protein synthesis in the hour following the stimulation. The rate of protein synthesis in preparations incubated with glucose alone in the medium was significantly higher than in preparations receiving either CHX or IAA. This occurred for both unstimulated and stimulated preparations. When incubated in media containing glucose alone, preparations that had been stimulated (3.1 ± 0.5% day^–1^) showed a significant increase in the rate of protein synthesis by 24% over control unstimulated preparations (2.5 ± 0.4% day^–1^). There was no difference in the rate of protein synthesis between unstimulated and stimulated preparations when the media contained either CHX or IAA.

### Metabolite Levels During Recovery From Simulated Jetting

**FIGURE 4:**
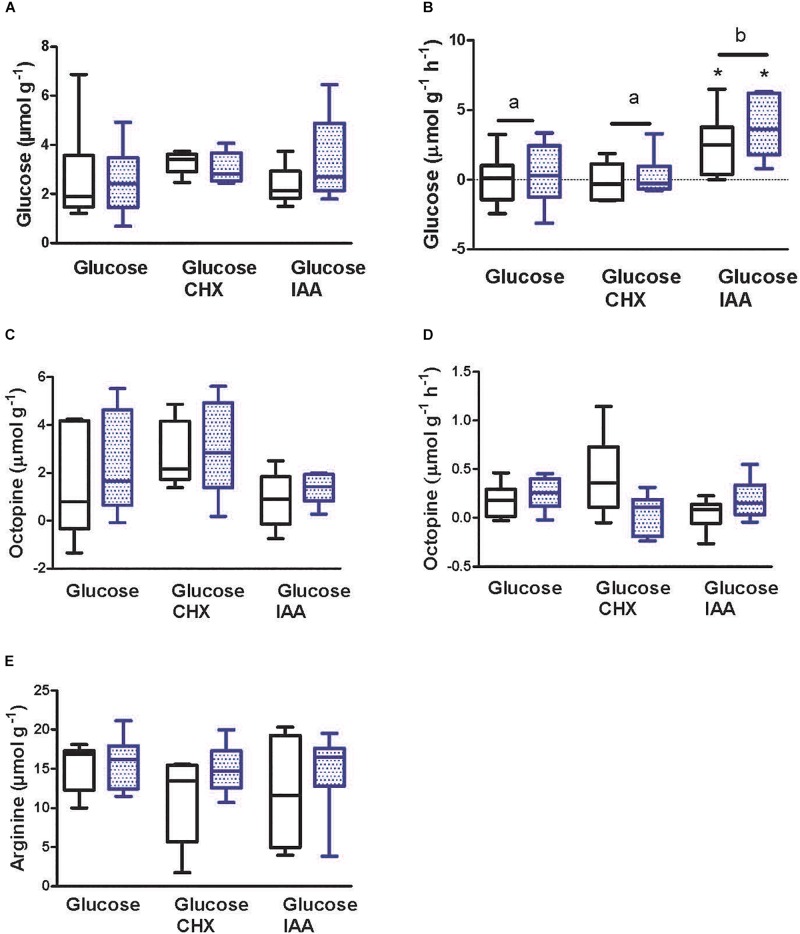
Glucose, octopine, and arginine levels following 1 h recovery from intense contractility. One of two paired mantle muscle sheets from cuttlefish (*Sepia officinalis*) was stimulated to contract for 30 s at 3 Hz simulating jetting behavior. **(A,C,E)** tissue concentrations after a 1 h period following contraction. **(B,D)** rate of uptake or release of metabolite into the bathing medium during the 1 h period following contraction. Open bars represent unstimulated preparations; stippled bars indicate preparations that were stimulated. Text below *x*-axis shows additions to media. For CHX and IAA additions, preparations were incubated for 10 min prior to stimulation, during stimulation, and throughout the post contraction incubation period. *N* = 6 under all conditions except tissue glucose in stimulated preparations incubated with CHX, tissue octopine in unstimulated preparations incubated with CHX, and media glucose in unstimulated preparations incubated with IAA where *N* = 5. Values with different letters are significantly different. ^∗^Significant difference from zero (one sample *t*-test) for rate of glucose appearance in bathing medium. Box and whisker plots show the range and median value.

Metabolite levels in mantle following the 1 h recovery period are shown in Figure 4. Glucose content of mantle sheets was approximately 3 μmol g^–1^ under all conditions (Figure 4A). There was no difference due to either stimulation or incubation condition in glucose content of mantle sheets. Mantle sheets were incubated in media containing 1 mmol L^–1^ glucose. There was no change in glucose concentration in the medium for preparations incubated with medium containing only glucose or in those containing glucose and CHX (Figure 4B). In preparations incubated with IAA in the medium there was a net increase in glucose in the medium (i.e., release from tissue) at a rate of approximately 3.3 μmol g^–1^ h^–1^. Eleven IAA-treated preparations (*N* = 5 unstimulated; *N* = 6 stimulated) showed an increase in glucose in the medium. Stimulation prior to the incubation period had no effect on glucose uptake or release.

Following 1 h of incubation, the mean level of mantle muscle octopine ranged from 1.43 to 2.99 μmol g^–1^. There was no significant difference in octopine content as a function of media composition or between unstimulated and stimulated preparations under any condition (Figure 4C). Octopine levels increased in the media during the incubation period under all conditions at mean rates of between 0.04 and 0.4 μmol g^–1^ h^–1^ (Figure 4D). There was no difference in the rate of octopine production as a function of stimulation or additions to the incubation media.

Following 1 h of incubation the mean level of arginine ranged from 12 to 16 μmol g^–1^ (Figure 4E). There was no significant difference in arginine content as a function of media composition. Also, stimulation had no effect on arginine level in paired preparations regardless of experimental condition. It was not possible to quantitate arginine phosphate with the method utilized; however, 28 of 36 preparations from the incubated preparations showed detectable arginine phosphate with a typical content of 3 μmol g^–1^. This value must be viewed with caution given the qualitative nature of the analysis and is likely an underestimate.

### Metabolite Levels Immediately Following Simulated Jetting

**FIGURE 5:**
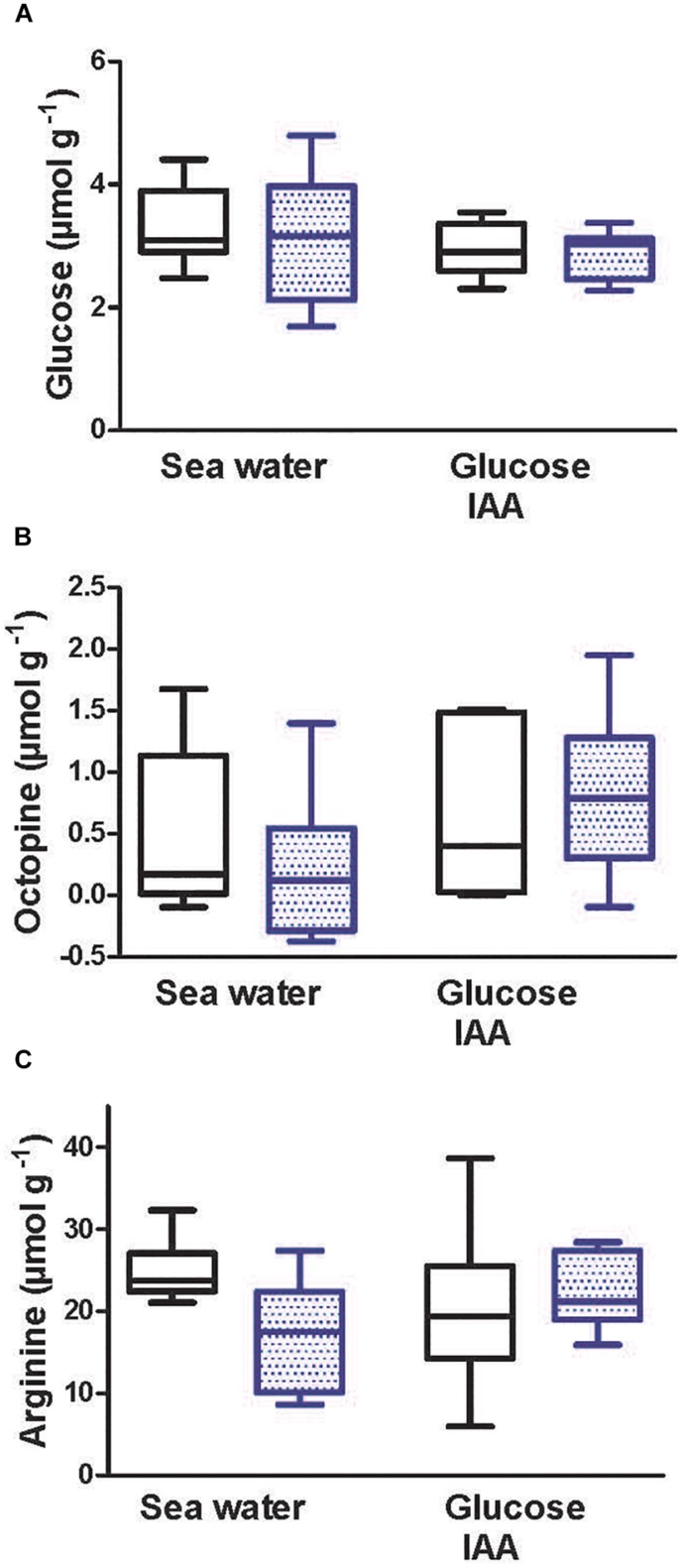
Glucose **(A)**, octopine **(B)**, and arginine **(C)** levels in mantle muscle sheets immediately following intense contractility. One of two paired mantle sheets from cuttlefish (*Sepia officinalis*) was stimulated to contract for 30 s at 3 Hz simulating jetting behavior and thereafter analyzed. Open bars represent unstimulated preparations; stippled bars indicate preparations that were stimulated. Text below *x*-axis shows additions to media. For IAA additions, preparations were incubated for 10 min prior to stimulation. *N* = 6 under all conditions. Box and whisker plots show the range and median value.

Metabolites were assessed immediately following the simulated maximum jetting contractions. The contractile challenge was similar to that in Figures 2C,D. One pair of mantle sheets was maintained in medium without additional glucose, the other pair received glucose plus IAA in the medium. Glucose content was approximately 3 μmol g^–1^ and did not change in different media or due to contraction (Figure 5A). Octopine content ranged from 0.21 to 0.82 μmol g^–1^ and did not change under any condition (Figure 5B). Tissue arginine content ranged from 17 to 24 μmol g^–1^ and similarly did not change under any condition (Figure 5C). Only 1 of 24 preparations gave a positive signal for arginine phosphate.

## Discussion

### Blockage of Protein Synthesis or Glycolysis Decreases Force Development Under Basal Conditions but Not Simulated Jetting Activity

The contractility experiment was designed to lend insight into mantle muscle physiology at both basal (i.e., low but not minimal) and high energy demand states. Force development during the initial hallmark challenge averaged a stress of 104 mN mm^–2^, in keeping with a previous study that reported peak contractile stress during tetanus of 226 mN mm^–2^ in similar preparations from *S. officinalis* of unknown but likely larger body mass, as animals were trawled off Plymouth, United Kingdom (Milligan et al., 1997). In concurrent assays, a number of preparations exhibited *F*_*max*_ values in excess of 300 mN mm^–2^ (data not shown). The current experiments were terminated after approximately 70 min but in parallel experiments force development could be sustained for at least a further hour. The preparation thus proved to be robust and suitable for metabolic studies.

Inclusion of CHX in the bathing medium resulted in a significant decrease in force following a 1 h recovery period after a simulated jetting event. One possible explanation for the loss in force is an impairment of synthesis of one or more proteins that have a high turnover rate and are necessary to support some aspect of contractility or provision of energy. Although the turnover rate of any specific protein, to our knowledge, has not been reported for any mollusk, a high rate of protein degradation may occur in *S. officinalis* mantle given its high calpain activity (Lamarre et al., 2012, 2016). Our interpretation is consistent with findings from a more well-studied muscle system, the perfused rabbit heart; hearts perfused with a cardioplegic medium prior to global ischemia show better contractile performance during normoxic reperfusion. The inclusion of CHX in the cardioplegic medium decreased that protective response and the protective effect of the cardioplegic medium is associated with a significant upregulation of a number of genes and proteins associated with contractility and energy metabolism (McCully et al., 2009). There was no significant decrease in stress development between control and CHX treated preparations either following the first 10 min rest period (during which time CHX was applied) or immediately after the high energy jetting challenge. It is possible that the time for CHX to take effect was too short to induce differences in protein turnover.

Inclusion of IAA in the bathing medium, under basal conditions (initial 10 min rest and 1 h rest following simulated jetting), resulted in a decrease in stress development relative to controls. Here, we accept that the primary impact of IAA is on glycolysis through inhibition of glyceraldehyde 3-P dehydrogenase although other effects independent of glycolysis are possible due to the non-specific mechanism of IAA on cysteine residues in proteins. With this caveat, we propose that an intact glycolysis during the recovery period is required to maintain subsequent contractile integrity. The impairment of contractility by IAA has been recognized in a variety of muscle types from different vertebrate species (e.g., frogs, Sandow and Karczmar, 1950; fish, Driedzic and Hart, 1985; rats, Ruff and Weissman, 1991). The relationship between glycolysis and contractility is discussed below. Glycolytic impairment however, had no impact on stress development following the 30 sec, simulated jetting challenge, suggesting that glucose metabolism is not a determinate of stress development during the high energy demand period.

Overall, the findings show that both an intact protein synthesis and glycolysis are required to maintain functional integrity during a 1 h recovery period following intense contractility. Under stimulated jetting conditions even control preparations lose stress but impairment of glycolysis does not exacerbate the response.

### Relationship Between Oxygen Consumption and Protein Synthesis Under Basal Conditions

*Ṁ*O_2_, determined for whole animals was 25–50% of the values previously reported for *S. officinalis* from the same geographic location but with a body mass of approximately 50 g (Lamarre et al., 2016; Capaz et al., 2017). A higher *Ṁ*O_2_ was anticipated based on the allometric equation for animals ranging in mass from 15 to 494 g (Melzner et al., 2007) however, weak allometric scaling between *Ṁ*O_2_ and mass was previously reported in smaller cuttlefish (Johansen et al., 1982). Differences in respirometry methods or size-dependent stress levels such as the body size/chamber volume may be critical, but it seems likely that the scaling relationship does not apply to small animals (Johansen et al., 1982). Indeed, there are other situations in cephalopods, such as in the Loliginidae family, that show little allometric scaling and *Ṁ*O_2_ is proportional to body mass (Seibel, 2007).

Regardless of the above comments, the *Ṁ*O_2_ reported here should be acceptable for further analysis of relationships within this particular experiment. The average rate of protein synthesis of isolated, unstimulated mantle was 2.51% day^–1^. This rate is the same as that found in the mantle of whole animals with a body mass of 50 g (Lamarre et al., 2016). Again higher rates of protein synthesis were expected in the smaller animals used in this study based on the general premise of allometric scaling of protein synthesis observed in dumpling squid (*Euprymna tasmanica*) (Carter et al., 2009; Moltschaniwskyj and Carter, 2010). Once more, lower than anticipated rates of oxygen consumption and protein synthesis are self-consistent within this study and suggest a lack of strong allometric scaling of both in small cuttlefish.

Unstimulated mantle had a *Ṁ*O_2_ of 10.58 μmol g^–1^ h^–1^ and a 1 g cuttlefish has a total mantle mass of 0.28 g. Mantle tissue in a 1 g animal could therefore account for a *Ṁ*O_2_ of 2.96 μmol h^–1^. Whole animal *Ṁ*O_2_ was 5.47 μmol g^–1^ h^–1^. Therefore, in a 1 g animal, mantle could account for 54% of the basal level of oxygen consumption. Both *Ṁ*O_2_ and rate of protein synthesis were decreased by the presence of CHX. In unstimulated mantle *Ṁ*O_2_ was decreased by 76% while protein synthesis was decreased by a comparable level of 65%. The parallel findings provide strong support for the concept that the decrease in *Ṁ*O_2_ by CHX is primarily due to an inhibition of protein synthesis but we cannot rule out secondary responses. If mantle accounts for 54% of the whole animal *Ṁ*O_2_ and 76% of mantle *Ṁ*O_2_ is due to protein synthesis, then a minimum of 41% of the whole animal oxygen consumption is being used to fuel protein synthesis in mantle under basal conditions. The 41% may be an underestimate given that CHX treatment did not block protein synthesis entirely.

The cost of protein synthesis may be estimated from *Ṁ*O_2_ and the fractional rate of protein synthesis. The total protein content of *S. officinalis* mantle is 166 mg g^–1^ (Sykes et al., 2009). Based on a fractional rate of protein synthesis of 2.51% day^–1^, this equates to 0.17 mg protein g^–1^ h^–1^. The CHX sensitive *Ṁ*O_2_ was 8 μmol g^–1^ h^–1^ (i.e., 10.58–2.57). Therefore, the cost of protein synthesis was 50 μmol O_2_ mg protein^–1^. Values for marine invertebrates using the approach of inhibition with CHX range from 14 to 148 μmol O_2_ mg protein^–1^ (Fraser and Rogers, 2007). Our calculated value sits in the middle of this range lending credibility to the findings and indicating that the protein synthetic machinery in *S. officinalis* is similar to other marine invertebrates.

### Protein Synthesis Is Stimulated Following Contractility

In preparations incubated with just glucose in the medium, *Ṁ*O_2_ was 29% higher following recovery from contractility than in unstimulated mantle. Similarly, protein synthesis was 24% higher following contraction than in unstimulated preparations. The essentially 1–1 correlation suggests that, the increase in oxygen consumption was due primarily to increases in protein synthesis and not a repayment of an anaerobic energy debt. The post exercise increase in protein synthesis in *S. officinalis* mantle muscle is similar to that which occurs in mammalian muscle (e.g., Biolo et al., 1995; Phillips et al., 1997). In mammalian, skeletal muscle activity may be associated with damage to contractile fibers (Gibala et al., 1995). We speculate that following activity, in cuttlefish, aimed at escaping predators or conspecifics, mechanisms are in place to enhance protein synthesis in mantle perhaps to repair damaged muscle fibers.

In preparations incubated with CHX there was a tendency for *Ṁ*O_2_ to be higher in stimulated than control preparations but with equivalent rates of protein synthesis. The marginally higher *Ṁ*O_2_ may represent an oxygen debt associated with repayment of anaerobic energy metabolism (i.e., Arg-P and/or ATP).

### Necessity for Low Rates of Glycolysis Under Basal Conditions

Inhibition of glycolysis with IAA during the non-contractile incubation periods resulted in a decrease in stress development, *Ṁ*O_2_, and protein synthesis. This suggests that glucose is required to fuel NADH production for oxidative phosphorylation, which in turn would drive ATP synthesis to support contractility and protein synthesis. Extracellular glucose was not called upon under any of the incubation conditions, implying that glycogen reserves are utilized. Unfortunately, due to an analytical error, glycogen levels are not available. More recent studies show that with unstimulated preparations, incubated in media containing glucose plus taurine, the final glycogen level was 1.56 ± 0.7 μmol glucosyl units/g (*N* = 9) (Driedzic et al., unpublished). The difference in *Ṁ*O_2_ between preparations incubated with glucose alone versus those with glucose plus IAA is 8–9 μmol g^–1^ h^–1^. Based on the standard relationship of glucose + 6 O_2_ yielding 6 CO_2_ + 6 H_2_O this would require a rate of glycolysis of 1.33–1.5 μmol g^–1^ h^–1^. As such, even if the initial glycogen level was only 3 μmol glucosyl units/g there would be sufficient starting levels to fuel aerobic metabolism and a low level of octopine production. In addition to direct provision of ATP to the contractile apparatus, a further nuance of the relationship between glycolytic inhibition and stress development should be considered. There is abundant evidence, from well-studied mammalian heart and skeletal muscle systems, that glycolysis under aerobic conditions leads to preferential provision of ATP generated in the cytosol to fuel membrane bound enzymes to support Na^+^ and Ca^2+^ transients (Nakamura et al., 1993; Xu et al., 1995; Dizon et al., 1998; Losito et al., 1998; Okamoto et al., 2001; Sepp et al., 2014). We propose that, as in mammalian systems, glycolytic inhibition over a number of minutes could impair Na^+^–K^+^ ATPase resulting in a derangement of intracellular Na^+^ and secondarily Ca^2+^ balance or could act directly on Ca^2+^ regulation at either the sarcoplasmic reticulum or the sarcolemma and in doing so lead to contractile failure.

### Conundrum of Glucose Gradients and Transport

A further nuance to observations on glucose levels deserves consideration. Glucose measured in tissue prior to and following a 1 h incubation was approximately 3 μmol g^–1^ wet weight. If all of the tissue free glucose is considered to be in the intracellular water and if the water content is 80% (Sykes et al., 2009) then the concentration of glucose in intracellular space is approximately 4 mmol L^–1^. But *in vivo* glucose levels in hemolymph never exceeds 2 mmol L^–1^ (Lamarre et al., 2012). Furthermore, mantle sheets were bathed in media containing 1 mmol L^–1^ glucose. The concentration of intracellular glucose was thus higher both prior to and following tissue incubation. There is also the finding that IAA treatment resulted in an increase in media glucose. The questions are “how can such a high intracellular to extracellular glucose gradient be maintained” and “where is the increased glucose in the media coming from.” There is preliminary evidence for an active glucose uptake by mantle of the Japanese oyster (*Crassostrea gigas*) (Bamford and Gingles, 1974) and a Na^+^ coupled, active transport of glucose, occurs in frog skeletal muscle (Kitasato and Marunaka, 1985). Given that glucose uptake has been little studied in marine invertebrates (Martínez-Quintana and Yepiz-Plascencia, 2012), the possibility of active glucose uptake by mantle of *S. officinalis* should be considered. Impairment of glycolysis with IAA could lead to a negative cascade whereby membrane based Na^+^–K^+^ ATPase would be compromised, and in turn impairing the ability to maintain an elevated intracellular glucose level leading to a movement of glucose down its concentration gradient and an increase in media glucose. The question of the source of glucose appearing in the media with IAA treatment is more difficult to resolve. Glucose could be released from glycogen but this requires an active glucose 6-phosphase that was undetectable in mantle from older cuttlefish (Speers-Roesch et al., 2016) but present in low activities in white muscle of numerous finfish (Short and Driedzic, 2018). It may be that the enzyme is active in younger *S. officinalis*, as well.

### Anaerobic Octopine Production Occurs During Recovery Not Activity

Loss of stress development during simulated jetting was similar with or without IAA in the medium, whereas, glycolytic blockage impaired restoration of stress development during the ensuing 1 h rest period (Figure 2). In preparations sampled immediately after the stimulated jetting event there was maintenance of high levels of intracellular glucose and no increase in octopine in either control preparations or those with glycolytic blockage (Figure 5), revealing that anaerobic glycolysis is not activated during the 30 s intense contractile challenge. Further tentative insights are gained by a general comparison of information obtained from all preparations sampled immediately after intense activity and following the subsequent rest period. Excluding preparations treated with IAA, octopine content significantly increased from 0.34 ± 0.19 (*N* = 12) (Figure 4C, sum of open bars) to 2.36 ± 0.41 μmol g^–1^ (*N* = 23) (Figure 5B, sum of open bars) (*p* = 0.0016; *t*-test) during a 1 h incubation period without contractile stimulation.

The current results show that in early juvenile *S. officinalis* a single physiologically relevant burst of mantle activity does not result in the anaerobic production of octopine. This finding differs from the substantial increase in mantle octopine to 8.6 μmol g^–1^ noted in larger cuttlefish (75 – 135 g) that had been chased to exhaustion (Storey and Storey, 1979). At this point we do not know if increases in mantle octopine require greater energy demand than that applied here, such as repeated jetting events, or if other factors such as age/body size or blood borne signals are determinants of octopine production. Our findings with isolated mantle of *S. officinalis* are consistent with the response in adductor muscle of scallop (*Pecten maximus*); an activity response within the normal physiological window did not result in an increase in octopine production but octopine did accumulate when animals were forced to swim until exhaustion. Octopine further accumulated while in recovery for the first 10–20 min but returned to minimal levels after 12 h (Gäde et al., 1978). A similar pattern of octopine change was noted in another species of scallop (*Chlamys opercularis*) (Grieshaber, 1978).

Intense activity did not result in a change in arginine levels (Figure 5C). Again, based on an exercise challenge, it was anticipated that arginine levels would increase in association with a decrease in Arg-P (Storey and Storey, 1979). There was no change in arginine content between unstimulated and stimulated preparations following the rest period (Figure 4E). During the 1 h rest period, the pattern of arginine content was the mirror image of octopine levels; the rest period lead to a decrease in arginine content (again excluding IAA preparations) from 20.9 ± 1.94 (*N* = 12) (Figure 5C, sum of open bars) to 14.3 ± 0.8 μmol g^–1^ (*N* = 24) (Figure 4E, sum of open bars) (*p* = 0.0008; *t*-test). The method utilized here to detect Arg-P was only qualitative; that withstanding, we failed to detect Arg-P in preparations immediately following intense activity but following the rest period Arg-P was detected in most preparations at approximately 3 μmol g^–1^. Overall, the data suggest that Arg-P had discharged in all preparations, including unstimulated controls, prior to the *in vitro* challenges. This was presumably a response that occurred during capture, anesthesia, and dissection of mantle even though animals appeared to be unstressed and did not release ink. During the 1 h recovery period, Arg-P pools began to recover, in association with a decrease in free arginine. This is a common pattern observed in squid, cuttlefish and scallop during recovery from exhaustive activity (Gäde et al., 1978; Grieshaber, 1978; Storey and Storey, 1979; Pörtner et al., 1993).

## Conclusion

By measuring contractility, *Ṁ*O_2_, protein synthesis, and metabolite levels we are able to bring new insights into the integrative physiology of mantle muscle in early juvenile (≈1 g) *S. officinalis*. At this stage of the life cycle cuttlefish grow at a rate of 12% per day (Sykes et al., 2006, 2014) that is high even by cephalopod standards (Forsythe, 1993). The rapid growth rate is supported by the current finding that 41% of whole animal *Ṁ*O_2_ is used to support protein synthesis in mantle. The cost of protein synthesis in mantle is in the range reported for other aquatic animals, thus this component of growth is not elevated in *S. officinalis*. Under basal conditions an intact protein synthesis is required to maintain contractility possibly due to rapidly turning over proteins consistent with high calpain activity in mantle. A single jetting challenge stimulates protein synthesis by approximately 25% over a 1 h post contractile period. This response is similar to that which occurs in mammalian skeletal muscle. It may be that proteins are degraded during contractility perhaps to supply amino acids for ATP production, and that during recovery there is a compensatory protein synthesis. This compensated rate of protein synthesis appears to be mainly supported by a 30% increase of *Ṁ*O_2_.

Stress development, *Ṁ*O_2_, and protein synthesis are inferred to be supported by glycogenolysis in the isolated preparations but the use of endogenous amino acids should not be ruled out as the enzymatic profile of *S. officinalis* mantle reveals high levels of proteolytic activity, transaminases, and glutamate dehydrogenase (Speers-Roesch et al., 2016). Intracellular glucose is higher than can be accounted for by facilitated diffusion of glucose suggesting an active transport mechanism. Glycolysis is not activated and octopine does not accumulate during a single physiologically relevant exercise challenge; however, we cannot rule out that multiple challenges may activate octopine production.

## Data Availability

The datasets generated for this study are available on request to the corresponding author.

## Author Contributions

SL, TM, NC, JA, AS, and WD participated in all aspects of the study. ÉB and VP performed the experiments and conducted the data analysis. WD wrote the initial draft of the manuscript that was revised by other authors. VP and AS were responsible for the animal husbandry.

## Conflict of Interest Statement

The authors declare that the research was conducted in the absence of any commercial or financial relationships that could be construed as a potential conflict of interest.
